# Targeting phosphodiesterase 3B enhances cisplatin sensitivity in human cancer cells

**DOI:** 10.1002/cam4.56

**Published:** 2013-02-03

**Authors:** Katsuhiro Uzawa, Atsushi Kasamatsu, Takao Baba, Katsuya Usukura, Yasuhiro Saito, Kentaro Sakuma, Manabu Iyoda, Yosuke Sakamoto, Katsunori Ogawara, Masashi Shiiba, Hideki Tanzawa

**Affiliations:** 1 Department of Clinical Molecular Biology, Graduate School of Medicine, Chiba UniversityChiba, 260-8670, Japan; 2 Department of Dentistry-Oral and Maxillofacial Surgery, Chiba University HospitalChiba, 260-8677, Japan

**Keywords:** Apoptosis, cisplatin sensitivity, multidrug resistance, *phosphodiesterase 3B* gene

## Abstract

We previously reported that human squamous cell carcinoma (SCC) cell lines refractory to cis-diaminedichloro-platinum II (cisplatin [CDDP]) had significant upregulation of the *phosphodiesterase 3B* gene (*PDE3B*), suggesting that inhibiting PDE3B suppresses CDDP resistance. shRNA-mediated PDE3B depletion in CDDP-resistant cells derived from SCC cells and Hela cells and induced CDDP sensitivity and inhibited tumor growth with elevated cyclic GMP induction resulting in upregulation of the multidrug-resistant molecule, but this did not occur in the 5-fluorouracil-resistant hepatocellular carcinoma cell lines. Furthermore, the antitumor growth effect of the combination of a PDE3B inhibitor (cilostazol) and CDDP in vivo was also greater than with either cilostazol or CDDP alone, with a significant increase in the number of apoptotic and cell growth-suppressive cancer cells in CDDP-resistance cell lines. Our results provided novel information on which to base further mechanistic studies of CDDP sensitization by inhibiting PDE3B in human cancer cells and for developing strategies to improve outcomes with concurrent chemotherapy.

## Introduction

Although the antitumor effect of cis-diaminedichloro-platinum II (cisplatin [CDDP]) has been proven in a board range of malignant tumors, its usefulness is limited due to severe side effects and acquired resistance [1–4]. Increasing evidence highlights the role of reliable markers of CDDP resistance in various human tumors, including squamous cell carcinomas (SCCs) of the lung, cervix, esophagus, and the head and neck [5–8]. In addition, gene expression microarray technologies have associated tumor gene(s) expression with the CDDP response that serves as prognostic indicators of survival in lung cancer, ovarian cancer, oral cancer, osteosarcoma, and malignant melanoma [9–13].

Our recent microarray analysis combined with the ingenuity pathway analysis (IPA) identified genes (*LUM*,* PDE3B*,* PDGF-C*,* NRG1*, and *PKD2*) associated with significant increases in CDDP-resistant squamous cancer cells compared with CDDP-sensitive squamous cancer cells in a dose- and time-dependent manner [13]. Results with the *phosphodiesterase 3B* gene (*PDE3B*), the inhibitor of which augments apoptosis of chronic lymphocytic leukemia [14], emphasized the association with CDDP resistance based on canonical pathway analysis, suggesting that the *PDE3B* gene may hold the key to the molecular mechanisms of resistant human cancer that might be chemotherapeutic molecular markers for choosing the appropriate chemotherapy for this disease.

Cilostazol has been widely administrated for the patients with peripheral vascular disease. In addition, it has been reported that cilostazol pharmacologically/selectively inhibits PDE3 with decreasing cellular proliferation through the cyclic adenosine monophosphate (cAMP) activation [15]. Here, we propose that this chemical could also act as an enhancer for the CDDP effect.

This work supported these speculations, because PDE3B expression was associated with CDDP resistance in human cancer cells and also showed that inhibiting the *PDE3B* gene by gene silencing and cilostazol results in enhanced CDDP responses in various human cancer cell lines in vitro and in vivo.

## Materials and Methods

### Cell lines

The Sa3 cell line derived from human oral SCC was purchased from the RIKEN Bio-Resource Center (Tsukuba, Ibaraki, Japan). The HLF cell line derived from undifferentiated human hepatocellular carcinoma was obtained from the Japanese Cancer Research Resources Bank (Ibaraki, Osaka, Japan). The human CDDP-resistant cell lines (Sa3-R and Hela-R) were kind gifts from Shigeyuki Fujita (Wakayama Medical University, Wakayama, Japan). The establishment of 5-Fu-resistant human hepatocellular carcinoma-derived cell line (HLF-R10) has been described in detail previously [16]. They were grown in Dulbecco's modified Eagle medium/F-12 HAM (Sigma–Aldrich Co, St. Louis, MO) supplemented with 10% fetal bovine serum (Sigma) and 50 units/mL penicillin and streptomycin (Sigma).

### Reagents

Reagents used in this study were CDDP (Sigma), and cilostazol (Sigma).

### shRNA assay

The CDDP-resistant cell lines (Sa3-R and Hela-R) were stably transfected with 0.5 μg/mL PDE3B shRNA (shPDE3B) or the control shRNA (Mock) (Santa Cruz Biotechnology, Santa Cruz, CA) construct by Lipofectamine LTX and Plus Reagents (Invitrogen, Carlsbad, CA) in 24-well culture plates. After transfection, the cells with the stably transfected shPDE3B were isolated by a culture medium containing 1 μg/mL puromycin (Invitrogen). Two to three weeks later, viable colonies were transferred to six-well plates and grown in the medium mentioned above.

### mRNA expression analyses

Total RNA was extracted from the cell lines using TRIZOL reagent (Invitrogen). We performed real-time quantitative reverse transcriptase-polymerase chain reaction analysis (qRT-PCR) analysis with one method using a LightCycler 480 Probes Master kit (Roche Diagnostics GmbH, Mannheim, Germany). Primers were designed using the ProbeFinder qPCR assay design software, which is freely accessible at the Universal ProbeLibrary Assay Design Center (https://www.roche-applied-science.com/sis/rtpcr/upl/index.jsp?id=uplct_030000). The nucleotide sequences of gene-specific primers for qRT-PCR amplification were: *PDE3B* forward, 5′-AAA GGG GAT AGA AAA CTT AAC AAG G-3′; and reverse, 5′-CAG GTA GCA ATC CTG AAG TTC C-3′ (universal probe #73). All qRT-PCR analyses were performed using the LightCycler^®^ 480 PCR system (Roche). The reaction mixture was loaded onto a PCR plate and subjected to an initial denaturation at 95°C for 10 min, followed by 45 rounds of amplification at 95°C (10 sec) for denaturation, 60°C (30 sec) for annealing, and 72°C (1 sec) for extension, followed by a cooling step at 50°C for 30 sec. The transcript amounts for the target genes were estimated from the respective standard curves and normalized to the *glyceraldehyde-3-phosphate dehydrogenase* (*GAPDH*) (forward, 5′-AGCCACATCGCTCAGACAC-3′; reverse, 5′-GCCCAATACGACCAAATCC-3′; and universal probe #60) transcript amount determined in corresponding samples.

### Western blot analysis

Western blot analysis of PDE3B and MRP2 protein in Sa3-R and Hela-R cells transfected with shPDE3B was performed. shPDE3B- and Mock-transfected cells were washed twice with cold phosphate-buffered saline (PBS) and centrifuged briefly. The cell pellets were incubated at 4°C for 30 min in a lysis buffer (7 mol/L urea, 2 mol/L thiourea, 4% w/v CHAPS, and 10 mmol/L Tris pH 7.4) with a proteinase inhibitor cocktail (Roche). The protein concentration was measured using the BCA Protein Assay Kit (Thermo, Rockford, IL). Protein extracts were electrophoresed on 4–12% Bis-Tris gel, transferred to nitrocellulose membranes (Invitrogen), and blocked for 1 h at room temperature in Blocking One (Nacalai Tesque, Kyoto, Japan). The membranes were washed three times with 0.1% Tween-20 in Tris-buffered saline and incubated with goat anti-human PDE3B polyclonal antibody (Abnova Corporation, Taipei, Taiwan) and mouse anti-human MRP2 monoclonal antibody (Santa Cruz Biotechnology) overnight at 4°C. The membranes were washed again and incubated using an anti-goat and anti-mouse IgG (H + L) horseradish peroxidase conjugate (Promega, Madison, WI) as a secondary antibody for 1 h at room temperature. The membranes were detected using SuperSignal West Pico Chemiluminescent substrate (Thermo), and immunoblotting was visualized by exposing the membranes to ATTO Light-Capture II (ATTO, Tokyo, Japan). Signal intensities were quantitated using the CS Analyzer version 3.0 software (ATTO).

### Clonogenic survival assay

To assess the sensitivity to CDDP, we determined proliferation rates using the 3-(4, 5-dimethylthiazol-2-yl)-5-(3-carboxymethoxyphenyl)-2-(4-sulfophenyl)-2H-tetrazolium) (MTS) assay (Promega). The cells were seeded in each well of 96-well plates at 2 × 10^3^ cells/well with DMEM containing 10% fetal bovine solution with various drug concentrations for 72 h. Then, 20 μL of MTS reagent was added directly to the adherent cells. The cells were incubated for 2 h at 37°C and the absorbance was recorded at 490 nm using a Benchmark Plus Microplate Reader (Bio-Rad Laboratories, Hercules, CA). The 50% inhibitory concentration (IC_50_) was defined as a 50% reduction in optimal density in each test and fractional absorbance was calculated using the formula: percentage of cell viability = [(mean absorbance of the experimental wells) − (absorbance of the blank)]/[(mean absorbance of untreated control wells) − (absorbance of the blank)] × 100%. The significance of the difference between shMOCK (triangles) and shPDE3B (circles) is indicated by asterisks (*P <**0.05).

### Measurement of cAMP/cGMP

The levels of cAMP and cyclic guanosine monophosphate (cGMP) were measured (fmol/well) using the commercially available Amersham cAMP or cGMP EIA Biotrak System (GE Healthcare, Little Chalfont, UK). sh*PDE3B*- and Mock-transfected cells were cultured in 96-well plates at a concentration of 10^4^ cells/mL. According to the manufacturer's protocol, the samples were acetylated and quantified in triplicate using the EIA kit. The plate optical density was determined using a plate reader at 450 nm within 30 min. The appropriate number of cells was cultured in 96-well plates and stimulated with cilostazol (50 μmol/L) at 4°C for 24 h. The appropriate number of cells was cultured in 96-well plates and stimulated with cilostazol (50 μmol/L) at 4°C for 24 h. According to the manufacturer's protocol, the samples were acetylated and quantified in triplicate using the EIA kit.

### PDE3B inhibition and CDDP enhancement in vivo

To investigate the antitumor activity of cilostazol combined with CDDP, we used xenograft models in the two sets of cell lines (Sa3/Sa3-R and Hela/Hela-R). The cells (2 × 10^6^) were injected subcutaneously into the backs of female athymic nude mice, BALB/cAnNcrj-nu/nu, purchased from Charles River Japan Inc. (Yokohama, Kanagawa, Japan). The care and treatment of experimental animals were in accordance with institutional guidelines. When the volume of the transplantation tumor reached 100 mm^3^, the mice were assigned randomly into four treatment groups: control (*n* = 5), cilostazol alone (*n* = 5), CDDP alone (*n* = 5), and cilostazol combined with CDDP (*n* = 5). The mice were treated with CDDP (5 mg/kg intraperitoneally once weekly for 4 weeks) and/or cilostazol (0.3% cilostazol-containing MF-based diet; Oriental yeast, Tokyo, Japan). The control and CDDP-alone groups received the MF control diet. The longest perpendicular tumor diameters were measured using calipers on alternate days to estimate the tumor volume using the formula: 4π/3 × (width/2)^2^ × (length/2). The values represent the mean tumor size ± standard error of the mean (SEM) (*n* = 5/group). The asterisks indicate significant differences from CDDP treatment alone (*P <**0.05, **P <**0.01, respectively). We next determined the effects of cilostazol combined with CDDP on tumor cell proliferation and apoptosis. The tumor tissues from the control, cilostazol alone, CDDP alone, and combination groups were then resected, and they were divided into two parts, one of which was frozen immediately after careful removal of the surrounding tissues and stored at −80°C until analysis of PDE3B expression by Western blotting mentioned above; the second part was fixed in 10% formalin, and paraffin sections (4 μm) were prepared for hematoxylin and eosin staining and immunohistochemistry of proliferating cell nuclear antigen (PCNA), Ki67, and cleaved caspase3 assay. Briefly, after deparaffinization and hydration, the endogenous peroxidase activity was quenched by 30-min incubation in a mixture of 0.3% hydrogen peroxide solution in 100% methanol, after which the sections were blocked for 2 h at room temperature with 1.5% blocking serum (Santa Cruz Biotechnology) in PBS before reaction with rabbit anti-PCNA polyclonal antibody (1:200 dilution; Santa Cruz Biotechnology), rabbit anti-Ki67 polyclonal antibody (1:50 dilution; Santa Cruz Biotechnology), and rabbit anti-cleaved caspase3 polyclonal antibody (1:100 dilution; Cell Signaling Technology, Danvers, MA) at 4°C in a moist chamber overnight. Upon incubation with the primary antibody, the specimens were washed three times in PBS and treated with Envision reagent (Dako, Carpinteria, CA), followed by color development in 3,3′-diaminobenzidine tetrahydrochloride (DAB; Dako). The slides then were lightly counterstained with hematoxylin, dehydrated with ethanol, cleaned with xylene and mounted. Nonspecific binding of an antibody to proteins other than the antigen sometimes occurred. As a negative control, triplicate sections were immunostained without exposure to primary antibodies, which confirmed the staining specificity.

### Statistical analyses

Continuous variables were assessed for normality using Kolmogorov–Smirnov test. The statistical significance was evaluated using the Wilcoxon signed-ranks test or nonpaired Student's *t*-test, as appropriate. *P *<**0.05 was considered statistically significant. *P <**0.05 and **P <**0.01. The data are expressed as the mean ± SEM. Statistical analyses were performed using SPSS 17.0 software (SPSS Inc., Chicago, IL) or Microsoft Excel (Microsoft, Redmond, WA).

### Results and Discussion

#### PDE3B expression and CDDP sensitivity in vitro

**Figure 1:**
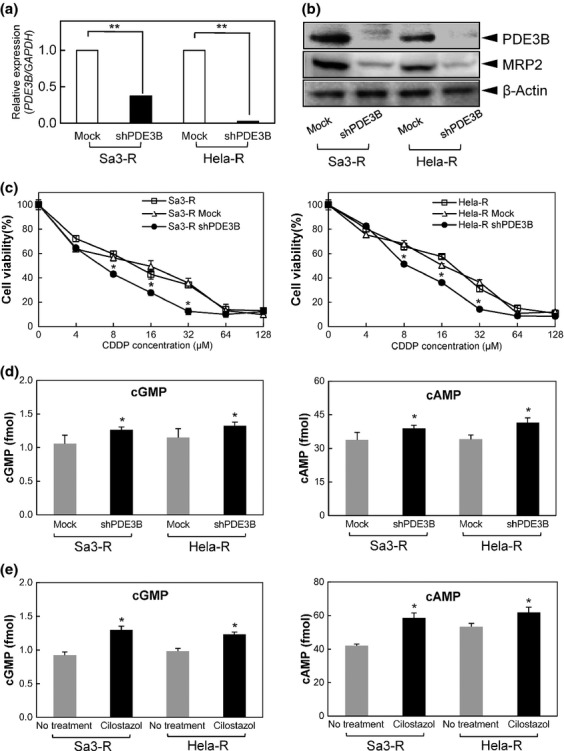
Analyses of *phosphodiesterase 3B* (*PDE3B*) inhibition in vitro. (a) Quantification of *PDE3B* mRNA expression in Sa3-R and Hela-R cells transfected with shPDE3B by quantitative reverse transcriptase-polymerase chain reaction (qRT-PCR) analysis. qRT-PCR shows that *PDE3B* mRNA expression in shPDE3B-transfected cells (Sa3-R- and Hela-R-derived transfectants) is significantly lower than in the shMock-transfected cells (**P <**0.01). (*b*) Western blot analysis of PDE3B and MRP2 protein in Sa3-R and Hela-R cells transfected with shPDE3B. Western blot analysis shows that the PDE3B and MRP2 protein levels in shPDE3B-transfected cells (Sa3-R- and Hela-R-derived transfectants) also have decreased markedly compared with that in the shMock-transfected cells. (c) Effect of cisplatin (CDDP) on the clonogenic survival of Sa3-R and Hela-R cells transfected with shRNAs. The results are expressed as the means ± standard error of the mean (SEM). The significance of the difference between shMock and shPDE3B is indicated by asterisks (*P <**0.05). (d) The levels of cyclic adenosine monophosphate (cAMP) and cyclic guanosine monophosphate (cGMP) in shPDE3B-transfected cells (Sa3-R- and Hela-R-derived transfectants). Both cAMP and cGMP levels in the PDE3B-knockdown CDDP-resistant cells are significantly more active than in the parental cells (*P <**0.05). (e) Effect of cilostazol, a selective PDE3 inhibitor, on cAMP/cGMP activation. To further investigate the mechanism by which reduction of PDE3B alters CDDP resistance, Sa3-R and Hela-R cells were exposed to cilostazol. Both CDDP-resistant cells had enhanced CDDP sensitivity in the presence of cilostazol with elevated cAMP/cGMP activation.

To exclude the possibility that the observed PDE3B linkage to CDDP resistance is restricted to a human SCC, we performed experiments in another human cervical carcinoma cell line (Hela) and a Hela-derived CDDP-resistant cell line (Hela-R) and compared the results with two human SCC-derived cell lines (Sa3/Sa3-R). Furthermore, to determine if PDE3B is associated with another type of antitumor drug resistance, we used a human hepatocellular carcinoma cell line with 10-fold greater resistance to 5-fluorouracil (5-FU) (HLF-R10) but not CDDP, and its parental cells (HLF) [16]. The IC_50_ values of CDDP for HLF and HLF-R10 cells were 81.6 μmol/L and 101.6 μmol/L, respectively. There was no significant difference in CDDP resistance between the 5-FU-resistant cells and the parent cells (Fig. S1a). By DNA short-tandem-repeat analysis (BEX Co. Ltd., Itabashi, Tokyo, Japan), the genetic fingerprinting using short-tandem-repeat markers of the two CDDP-resistant cell lines and HLF-R10 was authenticated with the three parental cell lines of identical origin, and the resistant cell lines were derived from one parental strain. To determine a causal link between CDDP sensitivity and *PDE3B* gene expression, mRNA expression was blocked in transfecting cells with PDE3B shRNA in the Sa3-R and Hela-R cells. Western blot analysis determined the PDE3B expression in the knockdown cells and nontarget control (shNT) cells (Fig. 1a and b). Although there was no significant difference in cellular proliferation between the shNT and knockdown cells, knockdown of PDE3B expression with 0.5 μg/mL PDE3B shRNA led to significant (*P <**0.05) abrogation of CDDP resistance in both CDDP-resistant cells (Fig. 1c) and restored the CDDP resistance to the level in the vehicle and shNT control. HLF and HLF-R10 showed faint PDE3B expression and no marked difference in expression level (Fig. S1b), indicating that PDE3B could play a specific role in CDDP resistance.

On the other hand, based on the accumulating data, a better understanding of robust specific biomarkers for CDDP resistance could be achieved. For example, a total of 1383 genes were listed up as aberrant expressed molecules in CDDP-resistant lung cancer cells [17], indicating that molecular mechanism of CDDP resistance consists of multifactorial processes including genetic/epigenetic alterations, aberrant gene expressions, and abnormal DNA repair. Furthermore, as described above, we have identified 199 genes as candidates for CDDP resistance in human oral SCC [13]. Thus, further studies are needed to test whether these genes are involved in the drug resistance in human cancers.

#### PDE3B regulation for cAMP/cGMP in CDDP-resistant cells

The main focus of the drug resistance of PDE3B has been metabolic regulation including insulin sensitivity regulated by cAMP and cGMP in adipocytes [18–20]. However, considerable evidence has suggested that cAMP and cGMP are crucial intracellular second messengers that regulate a wide variety of cellular processes including proliferation and apoptosis [21,22]. Aberrant regulation of these messengers might be an important system for CDDP resistance [23,24]. Moreover, among the known *PDE* gene families, PDE3B is the most abundantly expressed form in human leukemia cells [25]. We also found that cAMP and cGMP in the PDE3B-knockdown CDDP-resistant cells were more active than in the parental cells (Fig. 1d). In addition to cAMP/cGMP inhibition, significant downregulation of MRP-2 protein expression, mediated by cGMP [26], was detected in the knockdown cells (Fig. 1b). Our data and the other observations suggested a potential link between PDE3B overexpression, which results in MRP-2 upregulation through cAMP/cGMP degradation, and CDDP resistance in a series of human cancer cells.

#### Effect for CDDP enhancement with PDE3B inhibitor (Cilostazol) in vitro

Considerable evidence has indicated that a series of chemical inhibitors stop PDE3B expression [27]. We used cilostazol, a putative selective PDE3 inhibitor, in this study, because it has been used to treat human diseases [28–31]. Cilostazol increases the cellular levels of cAMP by inhibiting its degradation [32] and inhibits proliferation of the canine Madin–Darby cells [33]. To further investigate the mechanism by which reduction of PDE3B alters CDDP resistance, Sa3-R and Hela-R cells were exposed to cilostazol. As in PDE3B-knockdown cells, both CDDP-resistant cells had enhanced CDDP sensitivity in the presence of cilostazol with elevated cAMP/cGMP activation (Fig. 1e). We found no such improvement in the parental cells with a low mRNA level of the *PDE3B* gene. These data are consistent with the possibility that cilostazol might be a novel cancer drug that prevents PDE3B expression in CDDP-resistant cells. The 5-FU-resistant cells had no such chemical effect (Fig. S1b), suggesting that the effect of cilostazol may require aberrant expression of the PDE3B in tumor cells.

#### Effect for CDDP enhancement with PDE3B inhibitor (Cilostazol) in vivo

**Figure 2:**
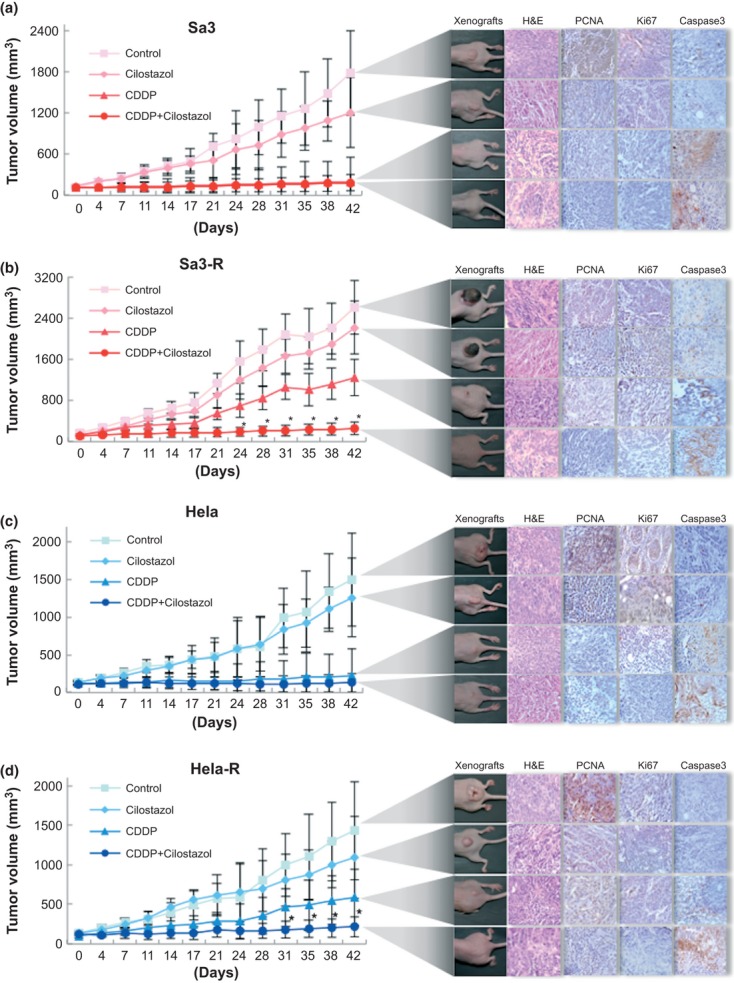
Analyses of PDE3B inhibition in vivo. Antitumor activity of cilostazol combined with cisplatin (CDDP) in the two sets of cell lines (Sa3 (a)/Sa3-R (b) and Hela (c)/Hela-R (d)) xenografts. The cells (2 × 10^6^) were injected subcutaneously into the backs of female athymic nude mice. When the volume of the transplantation tumor reached 100 mm^3^, the mice were assigned randomly into four treatment groups: control, cilostazol alone, CDDP alone, and cilostazol combined with CDDP. The values represent the mean tumor size ± standard error of the mean (SEM) (*n* = 5/group). The asterisks indicate significant differences from CDDP treatment alone (*P <**0.05). The tumor tissues from the control, cilostazol alone, CDDP alone, and combination groups were fixed in 10% formalin, and paraffin sections (4 μm) were prepared for hematoxylin and eosin staining and immunohistochemistry of proliferating cell nuclear antigen (PCNA), Ki67, and cleaved caspase3 assay. Original magnification ×400.

**Figure 3:**
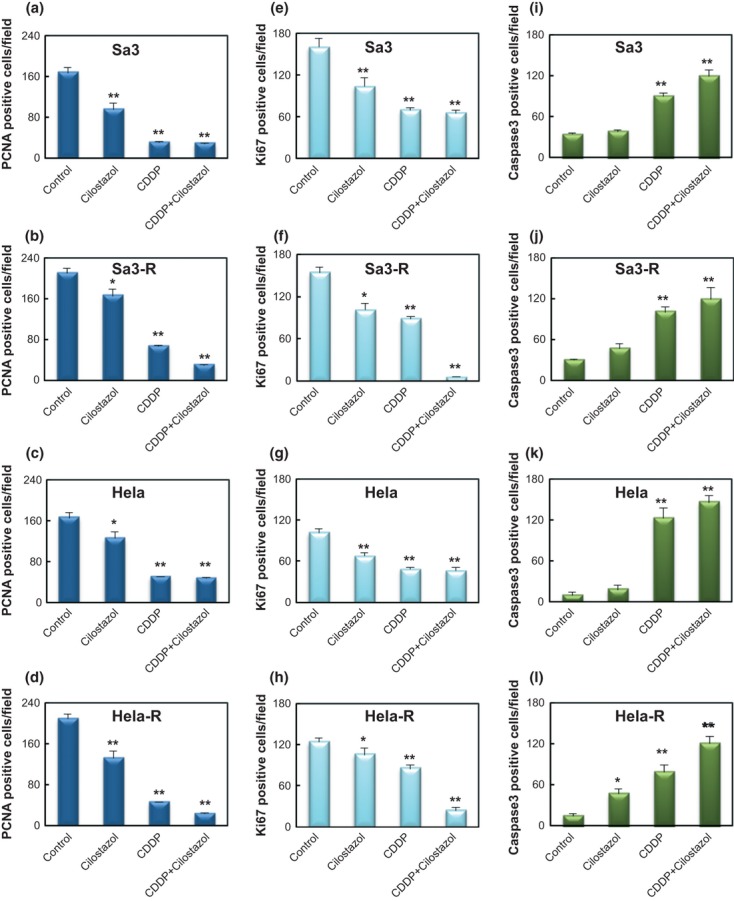
Quantification of proliferating cell nuclear antigen (PCNA)- (a–d), Ki67- (e–h), and caspase3- (i–l) positive cells. Results are expressed as the number of PCNA-, Ki67-, or caspase3-positive cells/field counted (five random fields per slide from a total of five slides per study group; magnification ×100). Data represent the mean ± standard error of the mean (SEM). The asterisks indicate significant differences from the controls (*P <**0.05, **P <**0.01).

**Figure 4:**
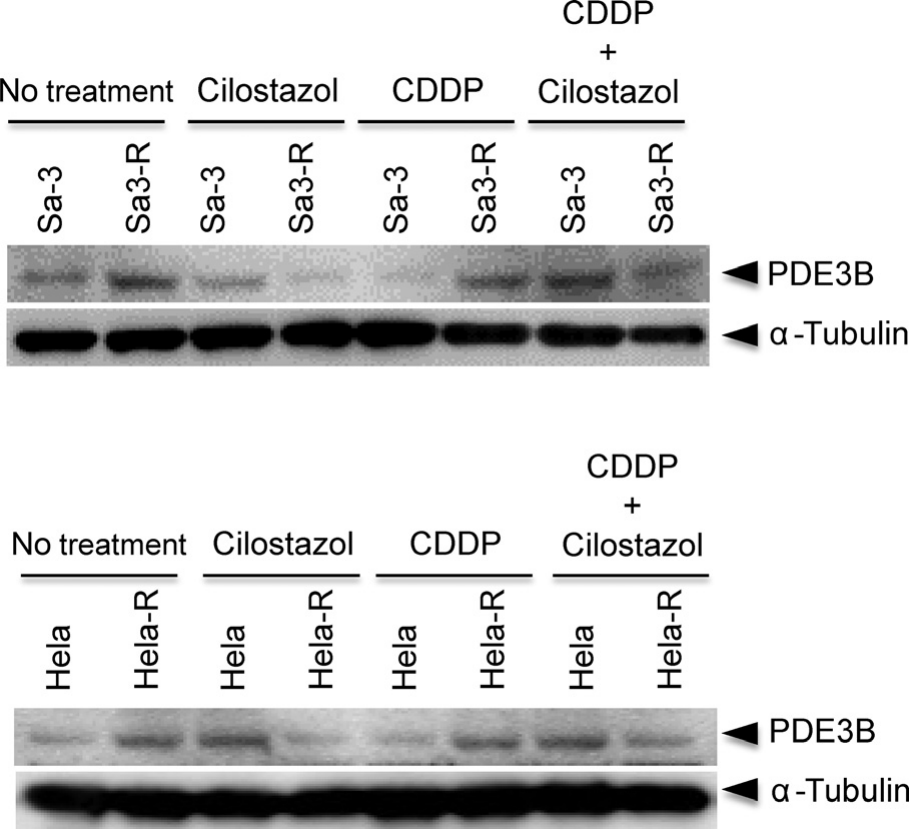
Effects of cilostazol on PDE3B downregulation in vivo. Note that the treatment of cilostazol is associated with a significant downregulation of PDE3B in tumors from cisplatin-resistant cells, but not in those from the parent cells. Equal loading was confirmed by stripping the membrane and reprobing them for α-tubulin and PDE3B protein levels.

**Figure 5:**
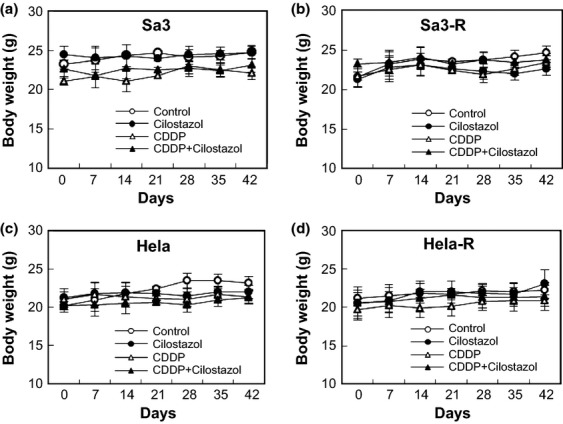
Comparison of the body weights of the control group and the cilostazol-treatment groups. The body weights were measured throughout the 42 days of treatment. Values shown represent the mean ± standard error of the mean (SEM) (*n* = 5 per group).

We then determined if cilostazol affects the tumor response to CDDP in vivo by evaluating the use of cilostazol to target tumor xenografts in mice. The Sa3/Sa3-R and Hela/Hela-R cells were inoculated subcutaneously into female athymic nude mice and grew to a mean volume of 100 mm^3^. The response of each target xenograft to CDDP plus cilostazol was enhanced significantly (*P <**0.05) only in the CDDP-resistant cells, compared with controls and systemic therapy with CDDP alone and cilostazol alone. In the parental cells, systemic cilostazol reduced the mean tumor volume by 32.2% (1212.3 ± 223.7 mm^3^ compared with 1785.6 ± 273.9 mm^3^ in the control group; *n* = 5) in Sa3 cells, and 16.1% (1258.4 ± 97.9 mm^3^ compared with 1499.6 ± 116.2 mm^3^ in the control group; *n* = 5) in Hela cells (Fig. 2c). CDDP only reduced the mean tumor volume by 89.0% (197.8 ± 21.2 mm^3^ compared with 1785.6 ± 273.9 mm^3^ in the control group; *n* = 5) in Sa3 cells and 85.5% (218.8 ± 44.5 mm^3^ compared with 1499.6 ± 116.2 mm^3^ in the control group; *n* = 5) in Hela cells (Fig. 2a and c). The effect of systemic cilostazol combined with CDDP did not differ for each single administration. In contrast, the resistant cells had consistently smaller tumors than control animals with combined treatments; the tumor volume decreased by about 90.0% (262.2 ± 56.1 mm^3^ compared with 2607.2 ± 276.4 mm^3^ in the control group; *n* = 5, *P *<**0.01) in Sa3-R cells, and 84.9% (215.9 ± 41.9 mm^3^ compared with 1430.0 ± 199.4 mm^3^ in the control group; *n* = 5, *P *<**0.01) in Hela-R cells (Fig. 2b and d). Because cellular proliferation and apoptosis can regulate tumor size at any given time, we performed immunohistochemistry of tumor tissues to measure the PCNA and Ki67 and cleaved caspase3 expression to measure apoptosis. Decreased proliferation and enhanced apoptosis were most pronounced in the combined-treatment group (average PCNA-, Ki67-, and caspase3-positive staining scores were 209.2 ± 6.4, 124.0 ± 2.9, and 16.2 ± 0.6 for the control; 132.8 ± 3.9, 105.4 ± 4.6, and 51.6 ± 0.9 for cilostazol alone; 46.0 ± 1.4, 85.2 ± 2.3, and 77.2 ± 2.2 for CDDP alone; and 23.6 ± 1.2, 7.0 ± 0.4, and 117.8 ± 1.4 for combination therapy; *n* = 5,*P <**0.05, **P <**0.01, respectively) for Hela-R cells (Fig. 3). Our findings suggested that the enhanced CDDP sensitivity resulting from combination CDDP and cilostazol are attributable partly to increased apoptosis and reduced tumor cell proliferation. It is noteworthy that while xenografted tumors of CDDP-resistant cells revealed a significant decrease in the level of PDE3B expression, those of the parent cells did not (Fig. 4). We also found that the average murine body weight in the cilostazol-treated group never decreased below that of the control group (CDDP only) (Fig. 5), indicating the novel/possible CDDP-based chemotherapeutic strategy for treating of human malignancies in clinical settings. This study did not provide direct evidence of a treatment benefit for PDE3B-expressing tumors, but cilostazol has been the most commonly used and well-tolerated drug for treating peripheral arterial disease [34], indicating the treatment potential.

In conclusion, development of CDDP sensitivity in human cancer cells involves reducing PDE3B. As the mechanism of CDDP resistance has been associated with multiple factors [35], numerous drugs that enhance CDDP sensitivity should be developed. To our knowledge, this report is the first on the combined use of a PDE3B inhibitor and CDDP in vitro and in vivo. The current data supported the idea that combination therapy of CDDP and cilostazol increases the therapeutic efficacy of CDDP-based chemotherapy in human cancers and could conceptually or strategically affect the design of future clinical trials for patients with cancer.

## Supporting Information

Figure S1. (a) Effect of CDDP on the clonogenic survival of HLF and HLF-R10 cells. A similar pattern of dose-dependent loss of cell viability in response to CDDP was observed between the 5-FU-resistant cells and the parent cells. The results are expressed as the means ± standard error of the mean (SEM) of the mean values from three assays. (b) Effect of cilostazol on *phosphodiesterase 3B* (*PDE3B*) expression of HLF and HLF-R10 cells. Western blotting revealed that the expression of PDE3B was unchanged by the treatment of cilostazol in the 5-FU-resistant cells and the parent cells. α-tubulin was used as the internal loading control.
