# Supplementary figures and images for: Targeting phosphodiesterase 3B enhances cisplatin sensitivity in human cancer cells
Source: Cancer Med. 2013 Feb 3;2(1):40–9. doi: 10.1002/cam4.56 (PMC3797561; doi:10.1002/cam4.56)

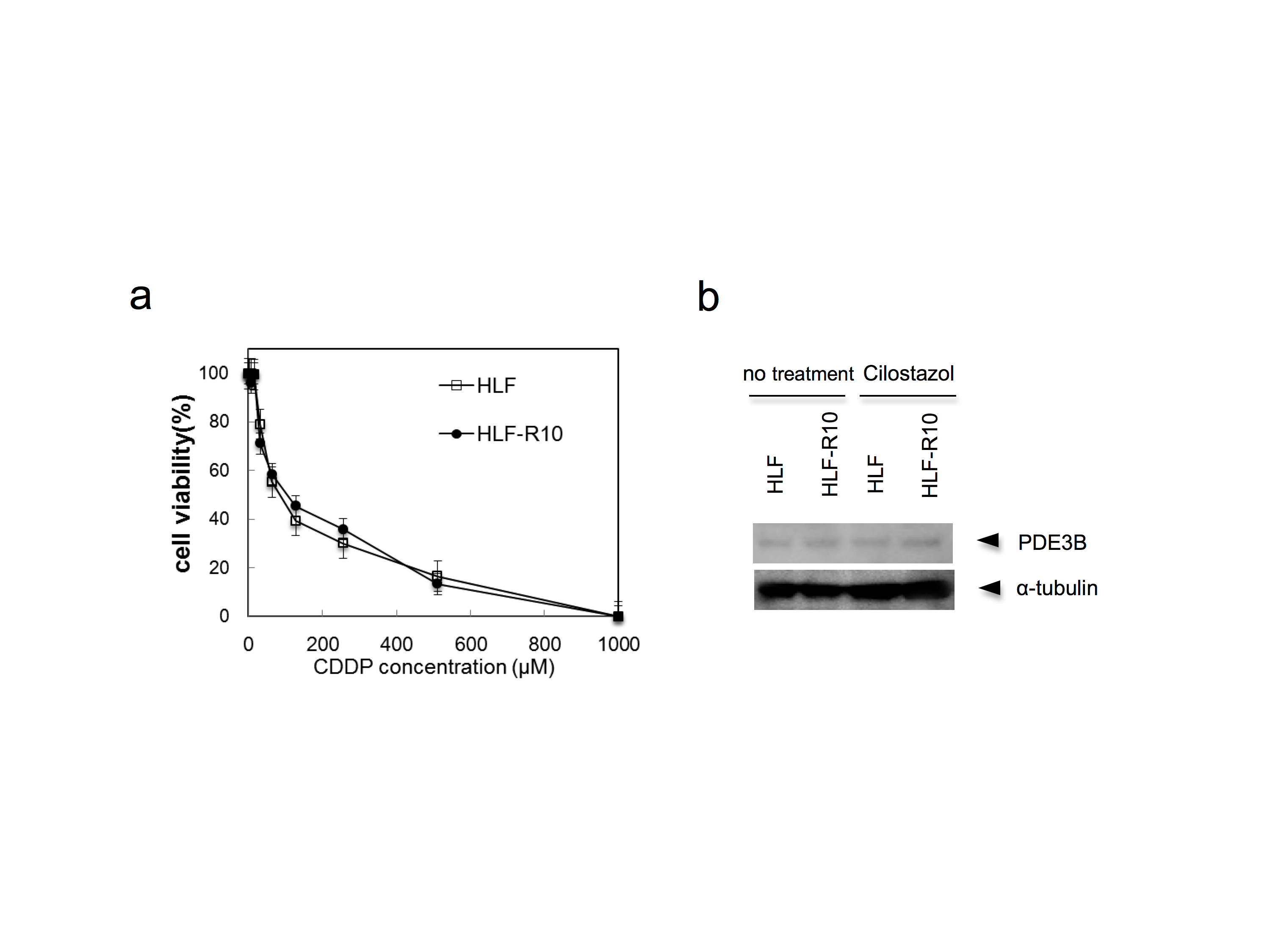

Supplement: Figure S1. — (a) Effect of CDDP on the clonogenic survival of HLF and HLF-R10 cells. A similar pattern of dose-dependent loss of cell viability in response to CDDP was observed between the 5-FU-resistant cells and the parent cells. The results are expressed as the means ± standard error of the mean (SEM) of the mean values from three assays. (b) Effect of cilostazol on phosphodiesterase 3B (PDE3B) expression of HLF and HLF-R10 cells. Western blotting revealed that the expression of PDE3B was unchanged by the treatment of cilostazol in the 5-FU-resistant cells and the parent cells. α-tubulin was used as the internal loading control. [file cam0002-0040-SD1.tif]
